# δ-Ctenitoxin-Pn1a, a Peptide from *Phoneutria nigriventer* Spider Venom, Shows Antinociceptive Effect Involving Opioid and Cannabinoid Systems, in Rats

**DOI:** 10.3390/toxins8040106

**Published:** 2016-04-12

**Authors:** Bruna Luiza Emerich, Renata C. M. Ferreira, Marta N. Cordeiro, Márcia Helena Borges, Adriano M. C. Pimenta, Suely G. Figueiredo, Igor Dimitri G. Duarte, Maria Elena de Lima

**Affiliations:** 1 Departmento de Bioquímica e Imunologia, Universidade Federal de Minas Gerais, Av. Antônio Carlos, 6627, Belo Horizonte, MG 31270-901, Brazil; brunaemerich@gmail.com (B.L.E.); apimenta@icb.ufmg.br (A.M.C.P.); 2 Departmento de Farmacologia, Universidade Federal de Minas Gerais, Av. Antônio Carlos, 6627, Belo Horizonte, MG 31270-901, Brazil; recmferreira@gmail.com (R.C.M.F.); dimitri@icb.ufmg.br (I.D.G.D.); 3 Fundação Ezequiel Dias, Rua Conde Pereira Carneiro, 80, Belo Horizonte, MG 30510010, Brazil; martadonascimento.phoneutria@gmail.com (M.N.C.); mhborgesb@gmail.com (M.H.B.); 4 Departamento de Ciências Fisiológicas, Universidade Federal do Espírito Santo, Av. Marechal Campos, 1468, Vitória, ES 29040-900, Brazil; suelygf@gmail.com

**Keywords:** spider toxin, δ-Ctenitoxin-Pn1a, PnTx4(6-1), *Phoneutria nigriventer*, spider venom, antinociception

## Abstract

PnTx4(6-1), henceforth renamed δ-Ctenitoxin-Pn1a (δ-CNTX-Pn1a), a peptide from *Phoneutria nigriventer* spider venom, initially described as an insect toxin, binds to site 3 of sodium channels in nerve cord synaptosomes and slows down sodium current inactivation in isolated axons in cockroaches (*Periplaneta americana*). δ-CNTX-Pn1a does not cause any apparent toxicity to mice, when intracerebroventricularly injected (30 μg). In this study, we evaluated the antinociceptive effect of δ-CNTX-Pn1a in three animal pain models and investigated its mechanism of action in acute pain. In the inflammatory pain model, induced by carrageenan, δ-CNTX-Pn1a restored the nociceptive threshold of rats, when intraplantarly injected, 2 h and 30 min after carrageenan administration. Concerning the neuropathic pain model, δ-CNTX-Pn1a, when intrathecally administered, reversed the hyperalgesia evoked by sciatic nerve constriction. In the acute pain model, induced by prostaglandin E_2_, intrathecal administration of δ-CNTX-Pn1a caused a dose-dependent antinociceptive effect. Using antagonists of the receptors, we showed that the antinociceptive effect of δ-CNTX-Pn1a involves both the cannabinoid system, through CB_1_ receptors, and the opioid system, through μ and δ receptors. Our data show, for the first time, that δ-Ctenitoxin-Pn1a is able to induce antinociception in inflammatory, neuropathic and acute pain models.

## 1. Introduction

The word pain derives from the Latin *poena*, which means “penalty” [1], and treating pain has always been a challenge to mankind since ancient times. Nowadays, the International Association for the Study of Pain describes it as “an unpleasant sensory and emotional experience associated with real or potential tissue damage”. Further understanding on the mechanisms by which pain signals are interpreted, transmitted and maintained will improve pain treatment strategies. 

A number of novel approaches using animal toxins to relieve pain have been investigated [2,3,4,5,6,7]. Indeed, peptides derived from animal venoms, including scorpions, spiders, amphibians, snakes and marine organisms, have been explored as antinociceptive agents. Many of them selectively inhibit voltage-activated Ca^2+^ and Na^+^ channels, acid-sensitive ion channels or glutamate ionotropic receptors [8,9,10,11,12] (for a review please refer to [3,5,6,7,13,14]). A successful example is the drug (Prialt^®^) derived from ω-conotoxin MVIIA, a peptide from *Conus magus* snail venom, known to have a pronounced analgesic effect resulting from the inhibition of voltage-activated Ca^2+^ channels [15,16]. 

It is also known that analgesia can be achieved by modulation of opioid and cannabinoid pathways [17,18]. Some peptides that have their mechanism of action based on this modulation have been characterized. One example is Crotalphine, an antinociceptive peptide based on the natural analgesic factor, isolated from the venom of the South American rattlesnake *Crotalus durissus terrificus* [19], that has its analgesic effect mediated by the release of peripheral dynorphin A, an endogenous agonist of κ-opioid receptors, and this release is dependent on cannabinoid receptor CB_2_ activation [20]. Some scorpion toxins, such as alpha-anatoxin Amm VIII, a weak modulator of Na_v_1.2 channel, and the depressant insect-selective beta-toxin LqqIT2, caused antinociception when injected in mammals, being the activation of endogenous opioid system suggested to be implicated [21]. Additionally, very recently, our group showed that a synthetic peptide called PnPP-19, derived from another toxin of the spider *Phoneutria nigriventer*, exhibits antinociception in rats via opioid and cannabinoid systems [22].

In the present work, we focused on δ-CNTX-Pn1a, a peptide isolated from the venom of the “armed” spider *Phoneutria nigriventer.* This spider is responsible for most of the envenomation cases in Brazil. Its venom is mainly composed of peptides, some proteins/enzymes, salts, amino acids and biogenic amines (for a review, please refer to [5,7,23,24]). Different peptides from this venom have been shown to target ion channels, as Na_v_ channels [25,26]; Ca_v_ channels [27,28,29], and K_v_ channels [30]. Another interesting group of peptides isolated from the venom of *P. nigriventer* comprises the insect toxins [31,32,33,34,35], which include PnTx4(6-1) or δ-CNTX-Pn1a, the toxin studied in this work, previously characterized to target insect sodium channels [9]. Herein we renamed this toxin, previously named PnTx4(6-1), taking into account the nomenclature proposed by King and co-authors [36], and we also highlight this new nomenclature for the other *P. nigriventer* peptides. δ-CNTX-Pn1a is a 48-amino-acid polypeptide, with 5 disulfide bridges (MW 5244.6 Da) [31]. The cDNA encoding this toxin was isolated and the toxin probably has a signal peptide followed by a propeptide [37]. This toxin binds to site 3 of sodium channels in cockroach nerve cord synaptosomes (*Periplaneta americana*) [9]. Indeed, although δ-CNTX-Pn1a slows down the inactivation of sodium currents in cockroach-isolated axons, it does not affect the currents in rat skeletal muscle (rNa_v_1.4/rSKM1) or brain (rNa_v_1.2/rBIIA) [9]. Moreover, at a high concentration (30 μg) it does not cause apparent toxicity to mice, when intracerebroventricularly (i.c.v) injected [31]. However, an action of this toxin in other sub-types of Na_v_s from mammals cannot be discarded, as we recently demonstrated for the recombinant PnTx4(5-5) (rΓ-ctenitoxin-Pn1a), another insect toxin from *P. nigriventer* venom, which shows 63% of identity with δ-CNTX-Pn1a [35]. rΓ-ctenitoxin-Pn1a was able to inhibit sodium currents on all mammalian Na_v_ channels tested (Na_v_1.2 to Na_v_1.6), with the highest current inhibition on Na_v_1.3 (38.43% ± 8.04%, IC_50_ = 1.5 μM). Despite its apparent lack of toxicity to mammals, we previously showed that Γ-ctenitoxin-Pn1a native reversibly inhibited the current of *N*-methyl-d-aspartate (NMDA) subtype of ionotropic glutamate receptors in rat hippocampal neurons [32]. In addition, we demonstrated that this toxin causes antinociception when peripherally injected in rats [38]. Additionally, it was recently shown that Γ-ctenitoxin-Pn1a promotes neuroprotection under insults of high levels of glutamate, in primary-cultured corticostriatal neurons from wild type mice, as well as from a mouse model of Huntington's disease [39]. It is well known that NMDA receptors are involved in nociception and that NMDA antagonists can produce antinociception [40,41].

Taking into account that δ-CNTX-Pn1a shows significant similarity with PnTx4(5-5) (Γ-ctenitoxin-Pn1a), which antagonized NMDA-receptor current in rat neurons and induced antinociception when peripherally tested in rats [38], this work aims at looking for the putative antinociceptive effect of δ-CNTX-Pn1a in different experimental pain models in rats: inflammatory, neuropathic and nociceptive. After confirming the antinociceptive effect of δ-CNTX-Pn1a in all these pain models, we proceeded to investigate the mechanisms underlying its analgesic properties, revealing the involvement of endogenous opioid and cannabinoid systems.

## 2. Results

### 2.1. Effect of δ-CNTX-Pn1a on Carrageenan-Induced Inflammatory Hyperalgesia

**Figure 1:**
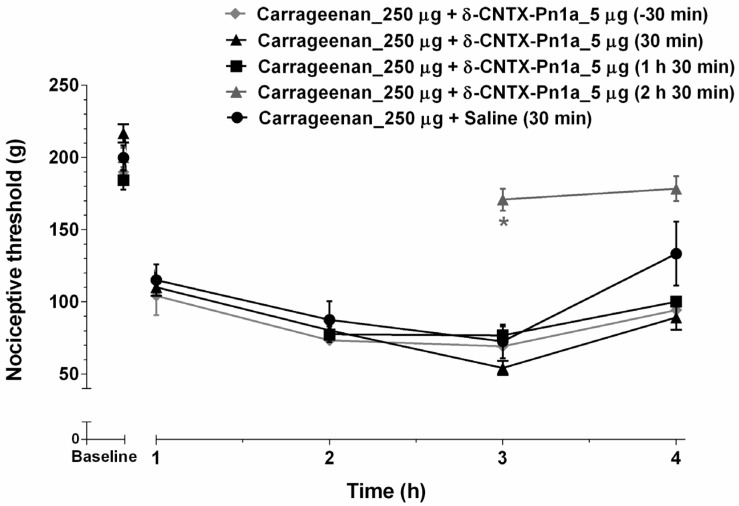
δ-CNTX-Pn1a antinociceptive effect following carrageenan-induced hyperalgesia. Rats were injected with 250 µg of carrageenan (Cg) into the right hind paw and then 5 µg of δ-CNTX-Pn1a (1 nmol) was intraplantarly administered into the same site, at different times (−30 min, 30 min, 1 h and 30 min, and 2 h and 30 min related to Cg injection). Nociceptive threshold was measured hourly 1 h after Cg injection. Each symbol represents MEAN ± SEM. *n* = 4 rats per group. Data were analyzed using ANOVA and Bonferroni post-test. *p* < 0.05 compared to Cg + Saline (*).

Administration of 5 µg of δ-CNTX-Pn1a, 2 h and 30 min after carrageenan (Cg), significantly increased the nociceptive threshold following carrageenan-induced inflammation. However, the administration of δ-CNTX-Pn1a by 30 min or 1 h and 30 min after Cg did not reverse hyperalgesia (Figure 1).

### 2.2. Effect of δ-CNTX-Pn1a on Neuropathic Hyperalgesia

**Figure 2:**
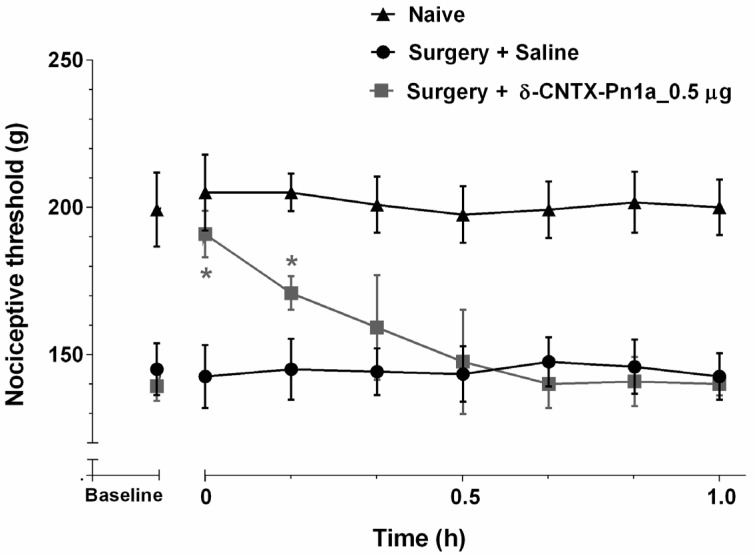
δ-CNTX-Pn1a antinociceptive effect on neuropathic hyperalgesia induced by surgical sciatic nerve constriction (14 days). Rats received 0.5 µg of δ-CNTX-Pn1a (0.1 nmol) or saline (control) through intrathecal injection. Rats that were not submitted to surgical intervention and not treated were labeled as naive. Nociceptive threshold was measured every 10 min, starting 5 min after the injections. Each symbol represents MEAN ± SEM. *n* = 4 rats per group. Data were analyzed using ANOVA and Bonferroni post-test. *p* < 0.05 compared to Surgery + Saline (*).

Intrathecal administration of 0.5 µg of δ-CNTX-Pn1a reversed the hyperalgesia induced by surgical sciatic nerve constriction. The analgesic effect was observed 5 min after administration and this effect progressively decreased with time, not being detected after 20 min (Figure 2).

### 2.3. Effect of δ-CNTX-Pn1a on Prostaglandin E_2_-Induced Hyperalgesia and Comparison between δ-CNTX-Pn1a and ω-Conotoxina MVIIA Antinociceptive Activity

**Figure 3:**
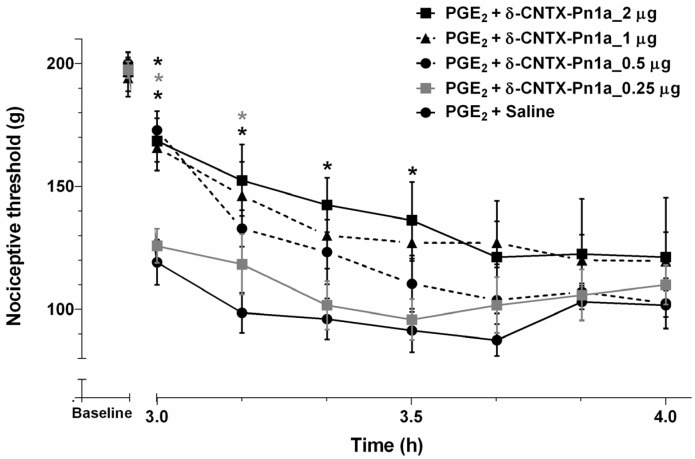
δ-CNTX-Pn1a antinociceptive effect following prostaglandin E_2_-induced hyperalgesia. Prostaglandin E_2_ (PGE_2_) (2 µg/paw) was administered through intraplantar injection. Rats received 0.25, 0.5, 1 and 2 µg of δ-CNTX-Pn1a (0.05, 0.1, 0.2 and 0.4 nmol) or saline (control) through intrathecal administration, 2 h and 55 min after PGE_2_ injection. Nociceptive threshold was measured every 10 min, starting 5 min after peptide or saline injections. Each symbol represents MEAN ± SEM. *n* = 4 rats per group. Data were analyzed using ANOVA, and Bonferroni post-test. *p* < 0.05 compared to PGE_2_ + Saline (*).

PGE_2_ is known to decrease the nociceptive threshold of nociceptors, exerting a critical role in the generation and maintenance of nociception [42]. We investigated the effect of δ-CNTX-Pn1a on PGE_2_-induced hyperalgesia and showed that this peptide increased, in a dose-dependent manner, the nociceptive threshold of rats. The highest tested dose (2 µg) induced a potent antinociception, which persisted for 30 min. This effect was less durable for the other tested doses (0.5 and 1 µg) (Figure 3).

**Figure 4:**
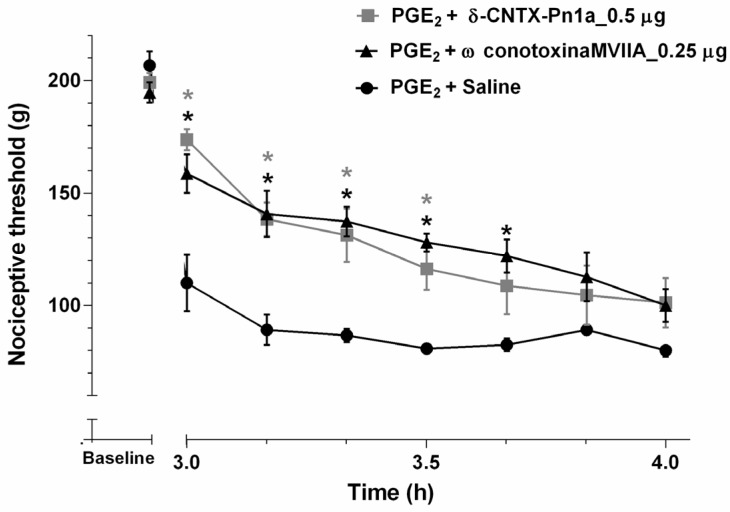
Antinociceptive effect of δ-CNTX-Pn1a and ω-conotoxin MVIIA following prostaglandin E_2_-induced hyperalgesia. Rats received 0.5 µg of δ-CNTX-Pn1a (0.1 nmol) or 0.25 µg ω-conotoxin MVIIA (0.1 nmol) or saline (control) through intrathecal administration 2 h and 55 min after intraplantar injection of prostaglandin E_2_ (PGE_2_) (2 µg/paw). Nociceptive threshold was measured every 10 min, starting 5 min after toxin or saline injections. Each symbol represents MEAN ± SEM. *n* = 4 rats per group. Data were analyzed using ANOVA and Bonferroni post-test. * *p* < 0.05 compared to PGE_2_ + Saline.

The antinociceptive effect produced by 0.5 µg (minimum effective dose) of δ-CNTX-Pn1a was compared to the antinociceptive effect of ω-conotoxin MVIIA on PGE_2_-induced hyperalgesia. The peptide (0.5 µg) was intrathecally administered and the nociceptive threshold was measured 5 min after the administration. The antinociceptive effect was similar for both peptides (Figure 4).

### 2.4. Involvement of Cannabinoid and Opioid Systems in δ-CNTX-Pn1a Antinociceptive Effect

**Figure 5:**
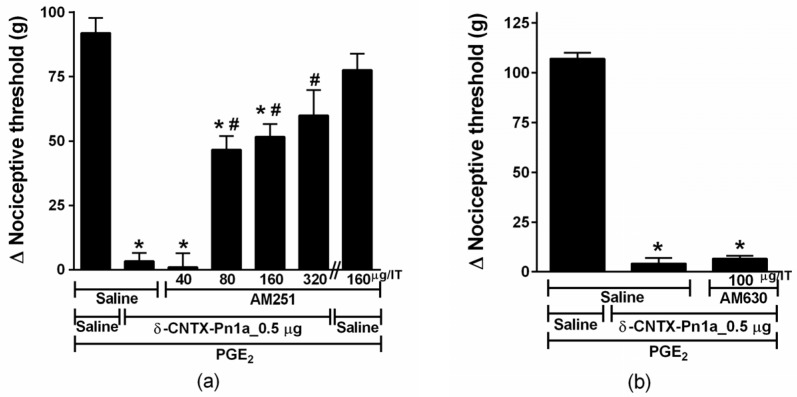
Effect of cannabinoid antagonists on δ-CNTX-Pn1a antinociception following prostaglandin E_2_-induced hyperalgesia. Rats received AM251 (**a**) or AM630 (**b**) through intrathecal route 2 h and 45 min after prostaglandin E_2_ (PGE_2_) injection (2 µg/paw). δ-CNTX-Pn1a, 0.5 μg (0.1 nmol), or saline (control) were intrathecally injected 10 min after the antagonists. Nociceptive threshold was measured 5 min after peptide or saline injection. Vertical bars represent MEAN ± SEM. *n* = 4 rats per group. Data were analyzed using ANOVA and Bonferroni post-test. *p* < 0.05 compared to PGE_2_ + Saline (*) or PGE_2_ + δ-CNTX-Pn1a + Saline (#).

The mechanism underlying δ-CNTX-Pn1a effect on the hyperalgesia induced by the inflammatory mediator PGE_2_ was also investigated. For this, we verified the possible effect of δ-CNTX-Pn1a on the cannabinoid and opioid pathways. In order to investigate the involvement of the cannabinoid system, animals were treated with AM251 (40, 80, 160 and 320 µg), a selective CB_1_ receptor antagonist, or AM630 (100 µg), a selective CB_2_ receptor antagonist, both administered 10 min before the peptide. AM251 reversed, in a dose-dependent manner, the antinociceptive effect of δ-CNTX-Pn1a, intrathecally administered (i.t.), on PGE_2_-induced hyperalgesia (Figure 5a). On the other hand, 100 μg of AM630 showed no significant effect on δ-CNTX-Pn1a antinociceptive effect (Figure 5b).

**Figure 6:**
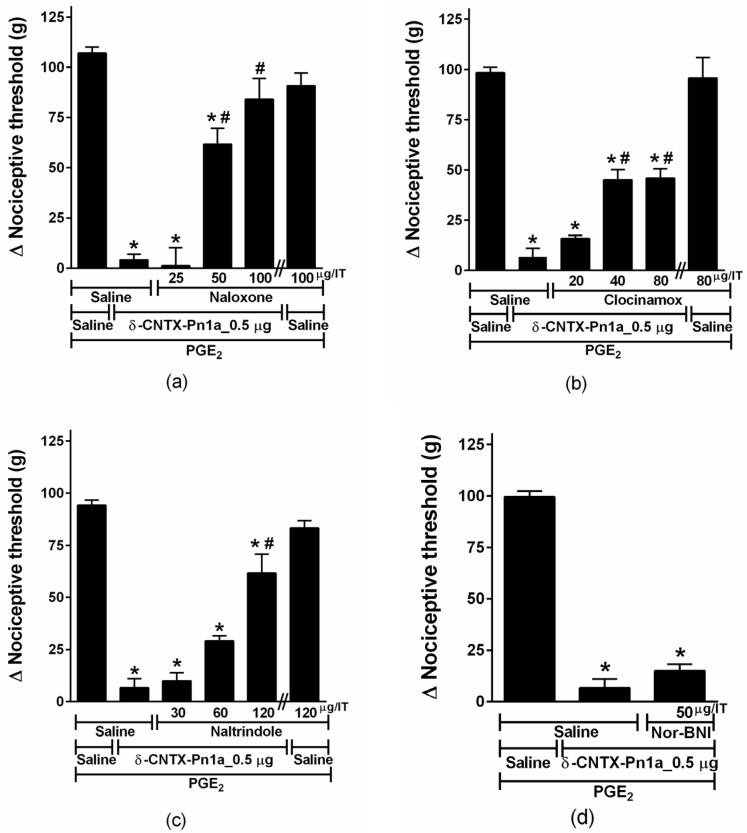
Effect of opioid antagonists on δ-CNTX-Pn1a antinociception following prostaglandin E_2_-induced hyperalgesia. Rats received Naloxone (**a**); Clocinamox (**b**); Naltrindole (**c**) or Nor-BNI (**d**) through intrathecal route 2 h and 25 min after prostaglandin E_2_ (PGE_2_) injection (2 µg/paw). δ-CNTX-Pn1a, 0.5 μg (0.1 nmol) or saline (control) were intrathecally injected 30 min after the antagonists. Nociceptive threshold was measured 5 min after toxin or saline injection. Vertical bars represent MEAN ± SEM. *n* = 4 rats per group. Data were analyzed using ANOVA and Bonferroni post-test. *p* < 0.05 compared to PGE_2_ + Saline (*) or PGE_2_ + δ-CNTX-Pn1a + Saline (#).

In order to explore the involvement of the opioid system, animals were treated with opioid receptor antagonists. The non-selective opioid antagonist, naloxone, reversed the antinociceptive effect of δ-CNTX-Pn1a (Figure 6a). Selective antagonists for μ (Clocinamox) and δ (Naltrindole) receptors significantly decreased δ-CNTX-Pn1a antinociception (Figure 6b,c). The selective antagonist for κ receptor (Nor-BNI) showed no significant effect on δ-CNTX-Pn1a antinociception (Figure 6d).

## 3. Discussion

The antinociceptive potential of animal toxins has been subject of several investigations that demonstrate the antinociceptive effect of venoms and many of their derivative peptides [5,6,12,43,44,45,46]. Our results show the analgesic effect of δ-CNTX-Pn1a in inflammatory, neuropathic and nociceptive *in vivo* pain models. Moreover, we reveal that the antinociceptive effect of δ-CNTX-Pn1a in the nociceptive pain model involves both opioid and cannabinoid endogenous systems.

Intraplantar administration of δ-CNTX-Pn1a inhibited inflammatory pain only when administered 2 h and 30 min after carrageenan injection. This effect may be related to the kinetics of mediators release in carrageenan-induced inflammation. In fact, many mediators are involved in carrageenan inflammation, including histamine, serotonin, kinins and prostaglandins [47]. The mediators involved in this nociceptive effect are most likely prostaglandins, which are known to decrease the threshold of nociceptor activation two hours after carrageenan administration [48]. 

The pharmacological treatment of neuropathic pain remains a challenge. Indeed, the current pharmacological clinical management of neuropathic pain achieves clinically significant pain relief in less than 50% of patients [49]. Here, we tested δ-CNTX-Pn1a in the neuropathic pain model and found that this peptide reversed the hyperalgesia caused by sciatic constriction. The effect of δ-CNTX-Pn1a was significant up to 10 min after injection, decreasing progressively over time, and losing effect after 20 min. 

According to Dalmolin and co-authors [46] and Oliveira and co-authors [50], two peptides isolated from the venom of *Phoneutria nigriventer* induce marked antinociceptive effect in an experimental model of neuropathic pain. Both peptides are able to modulate voltage-sensitive calcium channels: Tx3-3 (ω-ctenitoxin-Pn2a) blocks P/Q and R types, while Tx3-5 (U_7_-ctenitoxin-Pn1a) is a selective and potent blocker of L type. In low doses (3–300 fmol/site), Tx3-5 (U_7_-ctenitoxin-Pn1a) was able to produce antinociception in postoperative, neuropathic and cancer-related pain models. The analgesic potential of these toxins isolated from *Phoneutria nigriventer* venom seems evident. However, future studies using different concentrations of δ-CNTX-Pn1a may reveal better toxin-induced reversal of the hyperalgesia in our neuropathic model. 

It is known that loss of spinal opioid receptors and increased activity of physiological opioid antagonist systems occur in neuropathic pain. In contrast, no biologically relevant decrease in the number of CB_1_ receptors was evident after dorsal injury [49]. Although we did not investigate the mechanism of action of δ-CNTX-Pn1a in the neuropathic pain model, the effect of the peptide on CB_1_ receptors, observed for the nociceptive pain model discussed below, may encourage future investigations regarding a possible relationship between these receptors and the peptide analgesia observed in our neuropathic pain model.

When intrathecally administered, δ-CNTX-Pn1a showed a dose-dependent analgesic effect in PGE_2_-induced hyperalgesia. This effect was comparable to the antinociceptive effect of ω-conotoxin MVIIA, a peptide purified from the venom of the snail *Conus magus*. However, these two peptides apparently have distinct mechanisms of action. Indeed, the synthetic version of ω-conotoxin MVIIA is a powerful analgesic drug (Prialt^®^, Dublin, Ireland) that has a unique mechanism of action involving potent and selective blockage of *N*-type calcium channels [16]. On the other hand, the mechanism underlying the analgesic effect of δ-CNTX-Pn1a involves both cannabinoid and opioid receptors. It is well known that these receptors are involved in antinociceptive pathways. However, there are few studies regarding the effect of animal toxins on these receptors. Here, we show that only AM251, a selective CB_1_ receptor antagonist, when intrathecally administered, antagonizes the antinociceptive effect of δ-CNTX-Pn1a in PGE_2_-induced hyperalgesia (Figure 5a). Using naloxone, a nonselective opioid receptor antagonist, the antinociceptive effect of δ-CNTX-Pn1a was reverted (Figure 6a). In addition, we show that μ and δ opioid, but not κ receptors, are involved in δ-CNTX-Pn1a antinociception (Figure 6b,c). Pnpp19, a peptide synthetized by our group, in addition to potentiating erectile function in rats, showed antinocieptive activity against PGE_2_ hyperalgesia through activation of µ and δ opioid, and CB_1_ cannabinoid receptors. This peptide also seems to be able to indirectly induce antinociception through inhibition of a neuronal endopeptidase responsible for the cleavage of the endogenous opioid peptide encephalin [22]. Different from these findings, Crotalphine peptide reduced PGE_2_-induced hyperalgesia through an increased activation of both κ-opioid and CB_2_ cannabinoid receptors, being this effect mediated by dynorphin A [20]. This result reinforces the interaction between cannabinoid and opioid systems, as observed in our work and highlights the complexity of the nociceoptitive pathways. 

Finally, we recently showed that rPnTx4(5-5) (rΓ-ctenitoxin-Pn1a), another insect toxin from *P. nigriventer* showing 63% of similarity to δ-CNTX-Pn1a, obtained by heterologous expression in *E. coli*, besides strongly slowing down the inactivation of Bg Na_v_ (a sodium channel from *Blatella germanica* cockroach) also inhibited sodium currents on all tested Na_v_s from mammals (Na_v_1.2 to Na_v_1.6). It is worth noting that rΓ-ctenitoxin-Pn1a, as observed for δ-CNTX-Pn1a, did not show any apparent toxicity to mice, when i.c.v. injected (up to 30 μg). rΓ-ctenitoxin-Pn1a caused the highest current inhibition on Na_v_1.3 (38.43% ± 8.04%, IC_50_ = 1.5 μM) [35]. In addition to Na_v_1.3, some subtypes of Na_v_s, especially Na_v_s 1.7, l.8 and 1.9, have been described to play a significant role in pain processes. However, in order to investigate if the antinociceptive effect of these insect-toxins involves the inhibition of sodium channels, other assays are required, including the test of its effects on Na_v_s 1.7, 1.8 and 1.9, which are critically involved in pain mechanisms. Another possible target of these toxins, as observed for rΓ-ctenitoxin-Pn1a [38,39] is the NMDA receptor, which could also be involved in the observed antinociceptive effects. Other studies are in progress to try to clarify this point. 

## 4. Conclusions

In the present study, we reveal that δ-CNTX-Pn1a, a peptide isolated from the venom of *Phoneutria nigriventer* spider and previously characterized as an insect toxin, shows a clear analgesic effect in three different *in vivo* pain models. Moreover, we show, for the first time, the involvement of CB_1_ cannabinoid receptor and μ and δ opioid receptors in the antinociceptive effect of δ-CNTX-Pn1a. Taken together, our results may contribute for the development of novel therapeutic agents of a wide spectrum to manage pain, although studies are still necessary to better clarify the mechanisms involved in the effects of δ-CNTX-Pn1a.

## 5. Material and Methods

### 5.1. Animals

Male adult Wistar rats weighing 180–200 g were kept in a home cage environment with free access to water and food. Room temperature was maintained at 22 ± 2 °C with a 12–12 h light-dark cycle (lights on at 6:00 a.m.). Animals were acclimatized and familiarized with the experimental room for at least 1 day before testing. All experiments were carried out according to the current guidelines for the care of laboratory animals and ethical guidelines for investigations of experimental pain in conscious animals [51], and were approved by the Ethics Committee on Animal Experimentation of the Federal University of Minas Gerais (protocol number: 102/2012 from 4 July 2012).

### 5.2. Drugs

The following drugs and chemicals were used: δ-CNTX-Pn1a was purified by a combination of preparative reverse phase HPLC (RP-HPLC), ion exchange HPLC and analytical reverse phase HPLC as previously described [52]. µ-Conotoxin MVIIA was purchased from Latoxan (Valence, France). Carrageenan (Sigma, St Louis, MO, USA), Prostaglandin E_2_ (Enzo Life Sciences, Farmingdale, NY, USA), AM251 (*N*-(piperidin-1-yl)-5-(4-iodophenyl)-1-(2,4-dichlorophenyl)-4-methyl-1H-pyrazole-3-carboxamide; Tocris, Pittsburg, PA, USA), AM630 (6-iodo-2-methyl-1-[2-(4-morpholinyl)ethyl]-1H-indol-3-yl(4-ethoxyphenyl) methanone Tocris, Pittsburgh, PA, USA), Naloxone (Sigma, St. Louis, MO, USA), Clocinnamox (Tocris, Pittsburgh, PA, USA), Naltrindole (Tocris, Pittsburgh, PA, USA), Nor-BNI (Nor-Binaltorphimine dihydrochloride; Sigma, St. Louis, MO, USA) were dissolved as follows: PGE_2_ (2% ethanol in saline); AM251 and AM630 (12% DMSO in saline); Carrageenan, δ-CNTX-Pn1a, µ-Conotoxin MVIIA (MVIIA), Naloxone, Clocinnamox, Naltrindole and Nor-BNI (saline).

### 5.3. Drug Treatments

δ-CNTX-Pn1a (0.25–2 µg/site), MVIIA (0.25 µg/site), AM251 (40–320 μg/site), AM630 (100 μg/site), Naloxone (25–100 μg/site), Clocinnamox (20–80 μg/site), Naltrindole (30–120 μg/site) and Nor-BNI (50 μg/site) were administered through intrathecal (i.t.) route, according to Mestre and co-authors [53]. The i.t. injections were delivered in a volume of 20 µL/site per rat. δ-CNTX-Pn1a (5 µg/site), Carrageenan (250 μg/site) and Prostaglandin E_2_ (2 μg/site) were administered through intraplantar route (ipl.) in a volume of 50 or 100 μL.

### 5.4. Carrageenan-Induced Inflammatory Hyperalgesia

Male rats received a 100-μL intradermal injection of carrageenan (2.5 mg/mL in isotonic saline) into the right hind paw. δ-CNTX-Pn1a (5 µg/site) was administered through intraplantar route in different times after Cg injection. The nociceptive threshold was evaluated before and hourly after δ-CNTX-Pn1a administration.

### 5.5. Prostaglandin E_2_-Induced Nociceptive Hyperalgesia

Male rats received a 100 μL intradermal injection of PGE_2_ (0.02 mg/mL of PGE_2_, stored in ethanol, diluted in isotonic saline) into the right hind paw. δ-CNTX-Pn1a (0.25, 0.5, 1, 2 µg/site) was intrathecaly administered 2 h and 55 min after PGE_2_ injection. The nociceptive threshold was evaluated after δ-CNTX-Pn1a administration, every ten minutes.

### 5.6. Nociceptive Test

The nociceptive threshold was measured according to the rat paw pressure test described by Randall and Selitto [54]. We used an analgesimeter (Ugo Basile, Varese, Italy) with a cone-shaped paw presser with a rounded tip, which applies a linearly increasing force to the rat’s hind paw. The weight (g) required to elicit a nociceptive response (paw flexion) was determined as being the nociceptive threshold. A cutoff value of 300 g was used to minimize damage to the paws. The nociceptive threshold was measured in the right paw and determined as the average of three consecutive trials recorded before and various times after injection of the hyperalgesic agents. The same nociceptive thresholds were measured by two different experimenters. 

### 5.7. Induction of Neuropathy

For the induction of peripheral neuropathy, male rats were first anesthetized (60 mg/kg of ketamine plus 9 mg/kg of xylazine hydrochloride, intramuscular). Then, a partial constriction of the right sciatic nerve was performed using a previously described procedure [55]. In sham-operated rats, the control group, the nerve was only exposed without any ligation. Fourteen days after the surgical procedure the mechanical sensitivity was measured and, if the neuropathic pain effectiveness was established, the tests were performed. Rats not submitted to surgical intervention and not treated were labeled as naive. In this model of pain, δ-CNTX-Pn1a (0.5 µg/site) was intrathecaly administered [53].

### 5.8. Statistical Analysis

Data were analyzed for statistical significance by one-way ANOVA analysis of variance followed by Bonferroni’s test. The minimum level of significance considered was *p* < 0.05. All graphics and analyses were performed using Prisma 5.0. (GraphPad Software Inc, San Diego, CA, USA).

## Abbreviations

The following abbreviations are used in this manuscript:

δ-CNTX-Pn1a	δ-Ctenitoxin-Pn1a
NMDA	*N*-methyl-d-aspartate
Cg	Carrageenan
PGE_2_	Prostaglandin E_2_
AM251	*N*-(piperidin-1-yl)-5-(4-iodophenyl)-1-(2,4-dichlorophenyl)-4-methyl-1H-pyrazole-3-carboxamide
AM630	6-iodo-2-methyl-1-[2-(4-morpholinyl)ethyl]-1H-indol-3-yl(4-ethoxyphenyl)methanone
Nor-BNI	Nor-Binaltorphimine dihydrochloride
